# Nora Virus Persistent Infections Are Not Affected by the RNAi Machinery

**DOI:** 10.1371/journal.pone.0005731

**Published:** 2009-05-29

**Authors:** Mazen S. Habayeb, Jens-Ola Ekström, Dan Hultmark

**Affiliations:** 1 Department of Molecular Biology, Umeå University, Umeå, Sweden; 2 Institute of Medical Technology, University of Tampere, Tampere, Finland; Karolinska Institutet, Sweden

## Abstract

*Drosophila melanogaster* is widely used to decipher the innate immune system in response to various pathogens. The innate immune response towards persistent virus infections is among the least studied in this model system. We recently discovered a picorna-like virus, the Nora virus which gives rise to persistent and essentially symptom-free infections in *Drosophila melanogaster*. Here, we have used this virus to study the interaction with its host and with some of the known *Drosophila* antiviral immune pathways. First, we find a striking variability in the course of the infection, even between flies of the same inbred stock. Some flies are able to clear the Nora virus but not others. This phenomenon seems to be threshold-dependent; flies with a high-titer infection establish stable persistent infections, whereas flies with a lower level of infection are able to clear the virus. Surprisingly, we find that both the clearance of low-level Nora virus infections and the stability of persistent infections are unaffected by mutations in the RNAi pathways. Nora virus infections are also unaffected by mutations in the Toll and Jak-Stat pathways. In these respects, the Nora virus differs from other studied *Drosophila* RNA viruses.

## Introduction

The ultimate goal for viruses is to survive and propagate within its host. They do that either by infecting and propagating in a consecutive series of individual hosts, usually causing overt disease or death, or by infecting and persisting in a single host for a longer period of time. In the latter case, viruses need to infect the cells, manipulate the host replication machinery to replicate their genomes and get released from the infected cells without killing the host or triggering the immune system. Whereas some DNA- and retroviruses are able to persist by integrating their DNA into the host genome, it is still unclear how persistence is maintained by RNA viruses and by what mechanisms they evade the immune defense of the host.

In mammals, the innate and adaptive immune mechanisms act together to protect the host from viral infections. By contrast, the fruit fly *Drosophila melanogaster*, which lacks an adaptive immune system, depends entirely on the innate immune response. The absence of an adaptive immune response and the tractable genetics available in *Drosophila melanogaster* makes it a valuable model system to study the role of innate immunity in controlling viral infections.

It is still not fully understood how *Drosophila* controls viral infections. The Jak-Stat pathway has been shown to affect the course of *Drosophila* C virus infection [1], and there is some evidence for a role of the Toll pathway during an infection with the *Drosophila* X virus [2]. Best established is the role the RNAi machinery in the defense against RNA viruses [3], [4], [5], [6], [7].

The RNAi machinery uses small RNAs as guides to target complementary RNA and initiate the silencing of its expression. These small RNAs are divided into three groups: small interfering RNAs (siRNA), microRNAs (miRNA) and the piwi-associated interfering RNAs (piRNA) [8]. The siRNAs are directed towards viral or other exogenous double-stranded RNA, causing degradation of the complementary RNA. In contrast, miRNAs are directed towards endogenous mRNA and are involved in the regulation of mRNA expression. Finally, the piRNAs are important in the control of retrotransposons and are encoded in the germline of *Drosophila* [9].

In *Drosophila* two different RNase III enzymes, Dicer 1 and Dicer 2, process the double-stranded RNA to produce the miRNA and siRNA duplexes respectively [10]. Dicer-2 was shown to be a major factor in determining the outcome of an infection by several positive-strand RNA viruses: *Drosophila* C virus, flock house virus, cricket paralysis virus and the Sindbis virus [4], [6], [7]. The viral double-stranded RNA is cleaved by Dicer-2 into siRNAs of 21–23 nucleotides in length. The resulting siRNAs are then loaded onto a multiprotein complex, the RNAi-induced silencing complex (RISC), which contains a member of the Argonuate (AGO) family, AGO2, Vasa intronic gene (VIG) and potentially other associated proteins [11]. The guide strand of the siRNA, which bears complementarity with the target RNA, directs the highly specific cleavage of viral RNA molecules. Other components in the RNAi machinery have also been shown to play a role. For example, a mutation in R2D2, a double-stranded RNA-binding protein required downstream of Dicer-2, results in an increased flock house virus titer [6]. Flies deprived of essential components of the RNAi pathway also showed increased sensitivity to the *Drosophila* X virus, a double-stranded RNA virus. Moreover the virus appeared to replicate faster in cells deprived of AGO2 than in wild-type cell [3]. Recently it has been observed that Dicer-2 is also involved in sensing viral double-stranded RNA and inducing *Vago*, which participates in the control of viral titers in the fat body [12].

The best-characterized *Drosophila* viruses are all pathogenic, causing acute lethality or increased mortality in the infected stocks [2], [13], [14], [15], [16], [17]. In contrast, the Nora virus, a picorna-like virus recently identified in *Drosophila melanogaster*, causes persistent infections that are virtually asymptomatic [18], [19]. The Nora virus can therefore be used as a model to study the interaction of a persistent infection with the immune system of its host. In this report, we used the Nora virus to study the characteristics of the persistent infection and its interaction with the known anti-viral defenses in *Drosophila*. We observed a large variability in the ability of individual flies to clear the virus, depending on the viral titer in the infected fly. Surprisingly, we found that none of the known antiviral responses in *Drosophila* seems to play any role against the Nora virus.

## Materials and Methods

### Fly strains

All flies used were 3–5 days old and were reared at 25°C on standard yeast/agar media. Oregon R and Canton S flies were used as wild-type flies. The *AGO2^51B^* [20], *AGO2^414^* [21], *r2d2^S165fsX^* [6], [22], *Dcr-1^Q1147X^* and *Dcr-2^L811fsx^* [23], *piwi^1^* and *piwi^2^* [24], *hop^Tum^* [25] and *pll^2^* [26] fly stocks have been described previously.

### Viral infection protocol

Flies were infected by injection as described in [18]. Briefly, infected flies were homogenized in 1 ml NT buffer (100 mM NaCl, 10 mM Tris-HCl, pH 7.4), and clarified by centrifugation at 13,200×g for 5 min at 4°C. The supernatant was filtered through a 0.2 µm filter and the filtered preparation was used for infection of flies. Flies were anesthetized by CO_2_ and approximately 0.1 µl of the viral suspension was injected in the thorax. Flies were allowed to lay eggs during 7–10 days after the infection, and the viral titers were quantified in the offspring to confirm that we had established a permanently infected stock.

### RNA preparation and quantitative PCR

Total RNA was prepared using the Aurum total RNA kit (BioRad), according to the manufacturer. Quantitative RT-PCR was performed in duplicate, using the probe detection system in an I-cycle iQ Thermal Cycler (BioRad). The probe-based quantitative PCR was done with the following primers: forward, 5′-TTTCACTTTACTGTTGGTCTCC-3′; reverse, 5′-ATTCCATTTGTGACTGAT-TTTATTTC-3′ and a FAM/TAMRA probe: 5′-FAM-AGAGTTAGTGGACAA-GTTAGAGACTGGCAT-TAMRA-3′. cDNA synthesis was performed at 50°C for 10 min and 95°C for 3 minutes, followed by 40 cycles of amplification at 95°C for 10 seconds and 55°C for 30 seconds. The probe-based protocol works at an efficiency of 85%, and this value was used to calculate the relative concentration of viral RNA.

### Quantification of virus secretion in feces

To follow the viral production in single flies, we collected the feces of flies kept individually in 1.5 ml centrifuge tubes containing fly food. After 24 hours the flies were transferred to a new tube with fresh fly food. The same procedure was repeated daily during at least 19 days. We isolated viral RNA from the feces deposited in the tubes during days 1, 2, 3, 4, 7, 14 and 19. Total RNA was prepared using the Aurum total RNA kit (BioRad), after vortexing the feces deposited on the wall of the centrifuge tubes, with RNA extraction buffer (BioRad). For the clearance experiments, five days old single flies were kept in bottles and transferred twice per day into fresh bottles.

## Results

### Variability of Nora virus titers

**Figure 1:**
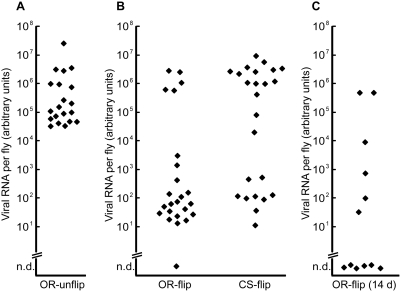
Nora virus clearance. Viral RNA in single flies kept (A) four days on contaminated food (unflip), or serially transferred for (B) four and (C) fourteen days to clean food (flip). Every dot represents the quantification from a single fly. OR: Oregon R, CS. Canton S, n.d: not detected.

We observed a large variability in the Nora virus titers between individuals of the same infected stock. The titers vary by three orders of magnitude [18] and Fig. 1a], suggesting that some flies may be able to clear the virus. To further investigate this possibility we minimized the re-infection via the fecal-oral route by keeping individual flies in separate vials, and transferring them twice per day to fresh food. After four days (Fig. 1b) or fourteen days (Fig. 1c), we quantified the viral RNA in these flies. We found that this treatment further enhanced the individual differences in viral titers. One population of flies retained the high titers of viral RNA, 10^6^–10^7^ arbitrary units, whereas a second population showed low or undetectable titers (typically 10–100 arbitrary units). Only few flies had intermediate titers. Similar results were seen with Oregon R (OR) and Canton S (CS) flies.

### Clearance of the Nora virus over time

**Figure 2:**
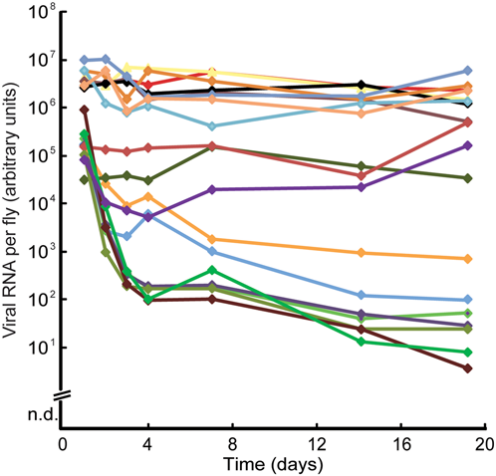
Clearance is threshold-dependent. Viral RNA secreted during 24 hrs in feces of single flies, serially transferred to clean food. Every line represents a single fly followed during 19 days. n.d: not detected.

We hypothesized that these individual variations in viral titers could be related to the replication cycle of the virus, causing bursts of viral production in the flies. To test this possibility, we next followed the virus production in single flies over time, by determining the virus titers in their feces. We transferred individual flies to fresh tubes with fly food every 24 hrs, and then quantified the viral RNA deposited in the tube during that time period (Fig. 2). This experiment clearly shows that there are no cyclic bursts of viral production. Instead, flies that deposited large amounts of virus on day one, also continued to produce virus at a relatively constant rate during all the examined days. In contrast, flies that produced less virus at the start of the experiment showed a dramatic further reduction in viral production during the first four days. Thereafter the viral deposition continued to decrease, but at a slower rate. A few flies were of medium titer, but they tended to either increase or decrease their viral production, approaching the high- or low-titer populations. This result excludes the possibility that the individual differences in virus titer are due to viral replication cycle. These results further demonstrate the persistence characteristics of the Nora virus, which is able to maintain a constant and high rate of viral production in some individuals, during a significant fraction of their life span. At the same time, other individuals appear to be able to fight the virus, although 19 days in this setting is not sufficient to clear the infection entirely.

### Nora virus and RNAi

The clearance of the virus in individuals of an infected stock, allowed us to study whether any of the known innate immune pathways are important in controlling the Nora virus. To study this question, we established Nora virus-infected stocks with mutations that affect these pathways. Such infected mutant flies were transferred to fresh food twice per day during four days, and then the viral titers in the flies was assayed to see the effect of the mutations on the clearance of the virus in low-titer infected flies, and on the persistence in the flies with a high-titer infection.

**Figure 3:**
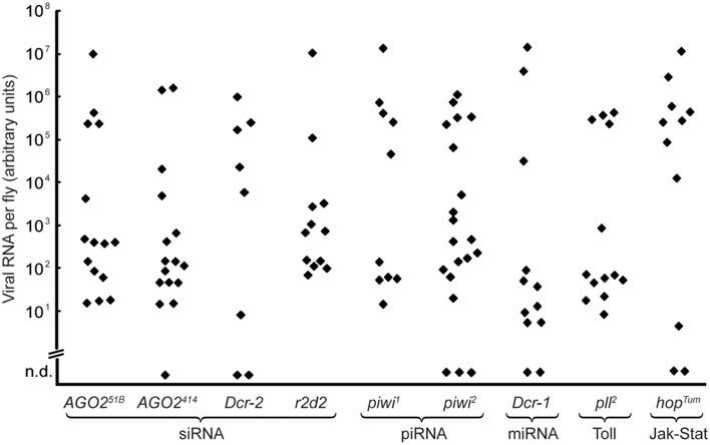
Nora virus control is siRNA-independent. Viral RNA was quantified in Nora virus-infected mutant flies, serially transferred for four days to clean food. *AGO2^51B^*, *AGO2^414^*, *Dcr-2^L811fsx^*, *r2d2^S165fsX^*, *piwi^1^ and piwi^2^*, are null homozygous mutants. *Dcr*-*1^Q1147X^* is a heterozygous null mutant. *hop^Tum^* is a constitutive active mutant and *pll^2^* is a strong loss-of-function allele. Every dot represents the viral load in a single fly. n.d: not detected.

We first tested homozygous null mutants of the *AGO2*, *Dicer-2*, and *r2d2* genes, which are all essential for the siRNA pathway and known to affect other *Drosophila* RNA viruses. Surprisingly, we observed no difference in the ability of these mutants to clear the Nora virus after an infection compared to the wild-type stocks (Fig. 3). For all mutants, a significant fraction of the individuals manage to clear the virus, fully or in part. Nor did we observe increased viral titers or any obvious lethality caused by the Nora virus in these mutants. From these experiments we conclude that the siRNA pathway does not significantly affect Nora virus persistence or clearance.

We also tested two different null mutants in *piwi*, which is involved in the control of retrotransposon RNA precursors via the piRNA pathway. Again, we saw no difference between piwi mutants and wildtype flies in their ability to clear the virus (Fig. 3). Finally, we also tested a null mutant in *Dicer-1* gene, which is important in the miRNA machinery that controls endogenous mRNA. *Dicer-1* is required for normal development, and the *Dcr-1^Q1147X^* mutant is therefore homozygous lethal, but at least in the heterozygous condition it does not affect the viral titers (Fig. 3).

### Nora virus and other immune pathways

Since the Toll and Jak-Stat pathways have also been implicated in controlling viral infections in *Drosophila*, we finally investigated if these pathways affect the Nora virus. For the Toll pathway we tested a strong loss-of-function allele in the *pelle* gene, *pll^2^*, which blocks Toll signaling when homozygous. However, that did not affect the ability of the mutant flies to clear the virus (Fig. 3). Thus, we conclude that the Toll pathway does not play a significant role in the interaction between the Nora virus and its host. Null mutants in the Jak-Stat pathway are homozygous lethal. Instead we tested a gain-of-function mutant, *hop^Tum^*, which has a continuously activated Jak-Stat pathway. However, Fig. 3 shows that *hop^Tum^* flies have normal Nora virus titers. That experiment does not exclude the possibility that Jak-Stat signaling might play a role, but it is clearly not sufficient to affect the Nora virus.

## Discussion

Our data show that the Nora virus is able to establish a persistent infection in the individual fly for a long period of time. This persistence seems to be determined by the virus titer in a threshold-dependent manner. A high-titer infection tends to stabilize at a high level, resulting in a persistent infection. On the other hand, flies with a lower titer seem to be more efficient in fighting the infection and reducing the initial viral load. Our experimental approach allowed us to test for two possible levels of Nora virus infection control. First, a mutation that inactivates the antiviral defense should result in flies that are unable to clear a low-titer infection. As a consequence, we expect all flies in the population to become persistently infected. Second, if the defense also limits viral replication in the persistently infected flies, we could expect increased viral titers and perhaps lethality. Surprisingly, none of these effects were observed for any of the mutants we tested. A fraction of the flies in all tested stocks were able to clear the virus, fully or in part, and another fraction reached a similar level of persistent infection as in the wild-type controls.

The lack of effect of the siRNA system was unexpected. This system is generally active against foreign replicating RNA and it is known to be essential in the defense against several RNA viruses in different organisms. It is possible that the Nora virus produces a suppressor of the siRNA-dependent RNAi machinery, or that it uses a stealth approach to avoid triggering the antiviral response. Viral suppressors of RNAi have been described from several viral systems [8], but we found no sequence in the Nora virus genome that is related to the viral suppressors that have been described previously. It should be noted however, that even the production of an RNAi inhibitor does not necessarily make a virus entirely resistant to the RNAi machinery, as shown for the flock house virus and the *Drosophila* C virus [6], [7]. These viruses remain partially inhibited by the machinery even though they encode RNAi inhibitors, as mutational inactivation of the RNAi machinery leads to increased viral loads and enhanced virulence. To our knowledge, there is no evidence of an RNAi inhibitor that could render the virus completely insensitive to the RNAi machinery. Further experiments are needed to determine whether the Nora virus encodes such an efficient inhibitor. We also failed to find any role for the two other main pathways of the RNAi machinery, or for Toll and Jak-Stat signaling. A caveat is that we could not test the null phenotypes of the developmentally essential miRNA and Jak-Stat pathways.

Although persistently infected flies produce Nora virus at a very high and stable rate, on the order of 10^8^ viral genomes per hour, viral replication is never so high that the survival of the host is endangered [18]. This balance might be influenced by defense mechanisms of the host, but the stability of the system is not in itself an argument for the existence of an antiviral defense in *Drosophila* against the Nora virus. If the virus carries its own mechanisms to limit replication, such a balance could be reached even in the complete absence of effective defenses. This situation would be ideal for the virus, where it would be able to evade the immune system and control its titers to levels that would maintain its presence by transmission to the next generation via the fecal-oral route. However, the clearance of the Nora virus from the low-titer flies is on the other hand a strong indication that defense mechanisms do exist in *Drosophila*, as it is unlikely that the virus encodes a mechanism for its own elimination.

The existence of both, a high variability in viral titers within the infected fly populations and a threshold titer that determines the persistence of the virus, makes us think that the immune system does interact with the Nora virus and influences the outcome of the infection. It remains to be determined by which mechanisms the flies clear the virus. To our knowledge this is the first example of an RNA virus that is completely unaffected by the inactivation of the RNAi machinery. It is also intriguing that the virus is not sensitive to any of the other immune pathways that have been shown to be important during an infection with other *Drosophila* viruses. Further genetic studies in *Drosophila* and in the Nora virus are likely to resolve these issues.
